# Determinants of Positive and Negative Affect among Adolescents and Young Adults in Indonesia: A Population-Based Survey

**DOI:** 10.3390/ijerph182312326

**Published:** 2021-11-24

**Authors:** Nurul Purborini, Ming-Been Lee, Hsiu-Ju Chang

**Affiliations:** 1 Department of Nursing, Faculty of Health Sciences, Muhammadiyah University of Magelang, Magelang 56172, Indonesia; ners.nurul@ummgl.ac.id; 2 Department of Psychiatry, National Taiwan University College of Medicine, Taipei 10617, Taiwan; mingbeen@ntu.edu.tw; 3 Taiwan Suicide Prevention Center, Taipei 10046, Taiwan; 4 Shin Kong Wu Ho-Su Memorial Hospital, Taipei 11101, Taiwan; 5 Department of Nursing, College of Nursing, National Yang Ming Chiao Tung University, No. 155, Section 2, Linong Street, Taipei 11221, Taiwan

**Keywords:** positive affect, negative affect, risk factors, adolescents, young adults

## Abstract

Positive and negative affect are crucial for mental health. However, the determinant factors of positive and negative affect have yet to be examined between adolescents and young adults. This study aimed to explore the determinant factors of positive and negative affect, comparing their effects among adolescents and young adults and among the two sexes in Indonesia. We undertook secondary data analyses of the Indonesia Family Life Survey for this cross-sectional study. Questionnaires on sociodemographic characteristics, physical and mental health-related variables, and childhood family experiences from 2014 were used as independent variables, and positive and negative affect were used as the dependent variables. Hierarchical linear regression was performed to investigate the factors associated with positive and negative affect and to compare their effects between adolescents and young adults. The hierarchical linear regression revealed that sociodemographic characteristics, perceived health, smoking, chronic condition, acute morbidity, sleep, childhood family experiences, depression, personality type, life satisfaction, happiness, and experience of disasters were associated with positive and negative affect among adolescents and young adults in Indonesia. Identification of positive and negative affect as well as their associated factors among adolescents and young adults should be considered when developing preventive programs in the community.

## 1. Introduction

Adolescence is a stage of life characterized by fundamental and rapid changes in an individual’s physical, cognitive, biological, social, and emotional system. The adolescent must cope with the changes, but the accompanying adaptation processes can be stressful [1]. Moreover, young adulthood is a crucial period of life because it represents the transition from adolescence to adulthood [2]. Young adults are obliged to meet the considerable expectations of surrounding influences, which can cause mental health problems or unhealthy behavior if they are unable to meet those expectations [3]. Adolescents and young adults are subject to high risk of anxiety and depression [4]. These problems occur because of physical transformations and emotional fluctuations that young people experience as a result of hormonal changes [5]. In such negative circumstances, positive emotion is important for young adults. Among young adults, positive mood is associated with the trait of resilience, and is a full mediator between the trait of resilience and positive meaning identification [6].

Emotions can be represented by two basic and distinct concepts of positive and negative affect. Positive affect (PA) is characterized by feelings of enthusiasm, vigor, and alertness, and negative affect (NA) by feelings of subjective distress and unpleasant engagement with one’s environment [7]. PA and NA possess some traits that reflect clear individual differences in positive and negative emotional experiences. Individuals who have stronger traits of NA are more prone to have NA and tend to worry excessively about threats and errors, whereas individuals who have high traits of PA are more prone to have PA and tend to be more confident and enthusiastic [8]. Traits of NA and PA are associated with personality traits, such as neuroticism and extraversion [9].

According to the broaden-and-build theory of positive emotion, positive and negative emotions have different and complementary adaptive functions, as well as cognitive and physiological effect. In this theory, negative emotions narrow a person’s mind–action repertoire by preparing a person to behave in a certain way. On the other hand, positive emotions (such as happiness, love, and satisfaction) expand a person’s mind–action repertoire, the range of emergent conditions, and behavioral thinking. As a result, these positive and negative emotions build an individual’s physical, intellectual, and social resources [10]. Many factors influence someone’s positive and negative emotions. These factors can be defined as risk factors and protective factors. Risk factors are characteristics (such as biological, psychological, or family) associated with a higher likelihood of negative outcomes. Meanwhile, protective factors are characteristics associated with a lower likelihood of negative outcomes. However, some risk and protective factors are fixed and others can change over time, such as income level, adverse childhood experiences, and employment status [11]. Among adolescents, risk factors could include female gender, early puberty, low self-esteem, anxiety, poor social skills, negative family environment, child abuse, family conflict, poverty, traumatic events, peer rejection, and poor academic achievement. For young adults, the risk factors could be early onset depression and anxiety, adverse childhood experiences, poor physical health, negative life events, decreases in social support, spousal conflict, and single parenthood [12]. 

PA has beneficial effects on cardiovascular function [13], overall physical health [14], and overall mental health [13]. Decreased PA can also act as a predictor for suicidal ideation. A study among women discovered that women exhibiting suicidal ideation had lower PA compared with women without such ideation [15]. PA is a part of positive experience and is mainly related to mood states [16]. In addition, improvements in sleep quality may be a predictor of increasing PA [17]. With the experience of more stressful events, PA tends to decrease [18]. Greater life satisfaction is related to higher PA [18,19]. Personality traits of extraversion and conscientiousness are positively associated with PA, whereas neuroticism is negatively associated with PA [8].

NA refers to the feeling of emotional distress [7], and when this feeling intensifies enough to disturb daily activity, it increases the risk of affective disorders, such as anxiety and depression. NA is also negatively correlated with general health and increases the risk of various health problems in later life, such as cardiovascular diseases, cancer, and type 2 diabetes [20]. Worse sleep has a positive association with NA [21], and improvement in sleep quality may predict decreasing NA [17]. The frequency and impact of stressful events had positive correlations with increases in NA [18]. Higher life satisfaction is related to lower NA [18,19]. In terms of personality traits, neuroticism is positively associated with NA [8].

One study discovered that a decreased ability to control PA may be associated with depression, whereas difficulty controlling NA is commonly associated with anxiety and depression [22]. Another study conducted in the Gaza Strip and West Bank, Palestine found that traumatic events decreased positive affect scores and increased negative affect scores in professional helpers (such as medical doctors, nurses, and social workers) [23]. Sex differences have also been observed in the prevalence of PA and NA. A study on risky driving reported that PA was a stronger predictor of risky driving in male drivers than in female drivers [24]. A cross-sectional study among Turkish high school students noted that female students had a higher mean score in NA, whereas male students had a higher mean score in PA [5]. However, limited studies on PA and NA among adolescents and young adults have been undertaken in Indonesia. A cross-sectional study of the general population in Indonesia discovered that dzikir (a spiritual ritual) could increase PA and reduce NA [25]. Thus, the aim of the current study was to identify particular sociodemographic characteristics, physical health-related variables, childhood family experiences, and mental health-related variables as determinant factors for PA and NA among adolescents and young adults, and to compare the predictive powers of these factors between the sexes. The principal hypotheses were (1) sociodemographic characteristics, physical health-related variables, childhood family experiences, and mental health-related variables are determinant factors for PA and NA among adolescents and young adults, and (2) there are different determinant factors for PA and NA between the sexes.

## 2. Materials and Methods

### 2.1. Design and Sample

This study used a cross-sectional design and a dataset from the Indonesia Family Life Survey (IFLS), which was conducted by the RAND Corporation (Santa Monica, CA, USA) in collaboration with SurveyMETER (Yogyakarta, Indonesia) [21]. The IFLS has followed the same individuals, households, and communities for >20 years. In 1993, the IFLS inquired into the population of 13 of 27 provinces in Indonesia, and adopted stratified sampling for distinct provinces and for rural or urban areas. Enumeration areas (EAs) were randomly selected in each province from a nationally representative sample. In the 13 surveyed provinces, 321 EAs were registered in 1993. In each of the urban and rural EAs, 20 and 30 households were selected, respectively. The sample represented approximately 83% of the Indonesian population, who lived in 321 EAs. Because the IFLS captures longitudinal data, the IFLS 2014 drew its sample from IFLS 1993, IFLS 1997, IFLS 2000, and IFLS 2007 [26].

### 2.2. Measurements

PA and NA were measured using an adapted version of the Positive and Negative Affect Schedule X (PANAS-X). The original PANAS-X has 20 questions, with 10 items related to PA and NA each [27]. After some adjustment to produce an Indonesia language version, the adapted PANAS-X used in this study had 12 items, covering both PA and NA, and was reported via responses on a Likert scale of 1 to 5. Those 12 items inquired into enthusiasm, frustration, sadness, contentment, boredom, tiredness, anxiety, loneliness, stress, anger, happiness, and pain [26]. Scores for PA and NA were calculated as the mean total scores [27]. In this study, the Cronbach’s α value for PANAS X was 0.79.

The independent variables in the study included sociodemographic characteristics, physical health-related variables, childhood family experiences, and mental health-related variables. The sociodemographic characteristics were age (15–18 y and 19–25 y), marital status (unmarried or married), sex (male or female), residential area (rural or urban), region (Java, Bali or other islands), school attendance status (no or yes), work status (no or yes), and economic status (poor, middle class, or wealthy). Physical health-related variables included perceived health, smoking habit details, smoking cessation details, chronic conditions, acute morbidities, and sleep status. Perceived health was assessed using the question, “In general, how is your health?” Smoking habit was assessed using the question, “Have you ever chewed tobacco, smoked a pipe, smoked self-rolled cigarettes, or smoked cigarettes?”, and smoking cessation was measured using the question, “Do you still have the habit or have you totally quit?” In this study, the following conditions were assessed by health care professionals and, if discovered, were considered to be chronic: hypertension; diabetes or high blood sugar; tuberculosis; asthma; other lung conditions; heart attack, coronary heart diseases, angina, or other heart problems; liver; stroke; cancer or malignant tumor; arthritis or rheumatism; high cholesterol (total or low-density lipoprotein); prostate illness; kidney disease (except for tumor or cancer); stomach or other digestive diseases; emotional, nervous, or psychiatric problems; and memory-related diseases. Acute morbidities included headache, runny nose, cough, breathing difficulty, fever, stomach ache, nausea or vomiting, diarrhea (minimal of 3 movements per day), swollen legs, skin infection, eye infection, toothache, and cold sores, which were experienced in the previous 4 weeks. Sleep was assessed using the Patient-Reported Outcomes Measurement Information System (PROMIS). The assessments were divided into sleep disturbance and sleep-related impairment. Each assessment module contained 5 questions and accompanying responses were given on a 5-point Likert scale. The total raw score was converted into T-score calculated as having a mean of 50 and a standard deviation (SD) of 10. Participants with a T-score of >50 were classified as having sleep disturbances or sleep-related impairment [28]. The Cronbach’s α for PROMIS was 0.82 in this study.

Childhood family experiences referred to self-rated childhood health and childhood experiences. Self-rated childhood health was assessed using the question, “Would you say that your health during your childhood was generally excellent, very good, good, fair, or poor?” Childhood experiences were assessed using four questions: (1) “When you were 12, were your biological parents still married?”, (2) “When you were 12, did you live with your biological mother?”, (3) “When you were 12, did you live with your biological father?”, and (4) “When you were 12, did you live with any of your grandparents?” In this study, the Cronbach’s α for this variable was 0.83.

Mental health-related variables included depression, personality type, life satisfaction, happiness, disaster experiences, and severe disaster experiences. Depression was measured using the 10-item Center for Epidemiologic Studies Depression Scale Revised (CESD-R 10). The 10 items were answered using a Likert scale of 0–3. Scores of ≥10 were considered to signify depression [29]. The Cronbach’s α for the CESD-R 10 was 0.72 in this study. Personality type was measured using the Big Five Index 15 (BFI 15). The BFI consists of 15 statements with every 3 statements covering 1 personality dimension; respondents replied to each statement on a Likert scale of 1–5. Scores for personality type were calculated using the mean total score for each of the personality dimension [30]. The Cronbach’s α for the BFI 15 was 0.81 in this study. Life satisfaction was measured using the question “Please think about your life as a whole, how satisfied are you with it?” Happiness was assessed using the question, “All things taken together, how would you say things are these day—would you say you are very happy, happy, unhappy, or very unhappy?” Disaster experiences was assessed using the question “In the last 5 years, was there any natural or other disaster (including civil strife) in the area where you live? If yes, what type of disasters?” Severe disaster experience was assessed using the question, “Were any of the disasters severe enough to cause death or major injuries to a household member, direct financial loss to the household, or a household member to relocate?”

### 2.3. Data Analyses

Stata version 13 was used for data analyses. Categorical and continuous variables were presented as the number (and accompanying percentages) of participants and means ± SDs, respectively. A Chi-square test was used to investigate the correlations of marital status, school attendance, economic status, residential area region, work status, perceived health, smoking habit details, smoking cessation details, depression, life satisfaction, happiness, acute morbidities, chronic conditions, physical activity, disaster experience, self-rated childhood health, and childhood experiences with age group and sex. An independent sample T-test was used to examine the correlations of personality type, PA, and NA with age group and sex. Hierarchical linear regression was performed to investigate the determinant factors of PA and NA among adolescents and young adults and different sexes. We developed four models for adolescents and young adults based on sex. In the first model, we put sociodemographic characteristics (marital status, attending school, economic status, residential area, region, and working status). For the second model, we added health-related variables (perceived health, smoking habit, smoking cessation, acute morbidities, chronic conditions, sleep disturbance, and sleep-related impairment). In the third model, we added childhood family experiences (self-rated childhood health, biological parents still married during childhood, living with biological mother during childhood, living with biological father during childhood, and living with grandparents during childhood). For the fourth model, we added mental health-related variables (depression, extraversion, conscientiousness, openness, neuroticism, agreeableness, life satisfaction, happiness, disaster experiences, and severe disaster experiences). Statistical significance was inferred when *p* < 0.05.

### 2.4. Ethical Considerations

The IFLS questionnaires and procedures were reviewed and approved by the Institutional Review Board of the RAND Corporation. In Indonesia, the IFLS received ethical clearance from Universitas Gadjah Mada. In Taiwan, the Joint Institutional Review Board of Taipei Medical University approved this study and provided the ethical clearance (reference no. N202009043). Written informed consent was obtained from all participants involved in the parent IFLS study. Personal records in the survey were kept anonymous and confidential [26].

## 3. Results

### 3.1. Characteristics of Study Participants

We analyzed data pertaining to adolescents and young adults aged 15–25 y who participated in the 2014 IFLS. In 2014, 34,389 participants were aged ≥15. Among all participants, 8532 (24.81%) were adolescents and young adults. All questionnaires were completed by 7589 (88.95%) adolescents and young adults.

Table 1 compares the participants based on age group and sex. In the bivariate analysis, the differences between male and female participants varied in the 15–18 y and 19–25 y groups. 

### 3.2. Factors Associated with PA

Table 2 displays the results of the hierarchical multiple linear regression of PA for the 15–18 y age group based on sex. Factors that were significantly associated with increasing PA among male adolescents included wealth (*B* = 0.227), happiness (*B* = 0.560), life satisfaction (*B* = 0.393), and openness (*B* = 0.062). However, living in other islands (*B* = −0.166), having depression (*B* = −0.160), neuroticism (*B* = −0.045), and having disaster experiences (*B* = −0.189) were associated with lower likelihood of having PA. 

Among female adolescents, happiness (*B* = 0.457), life satisfaction (*B* = 0.509), and openness (*B* = 0.065) were significantly associated with increasing PA. Conversely, living in other islands (*B* = −0.188) was associated with a lower likelihood of having PA.

Table 3 displays the results of the hierarchical multiple linear regression of PA for the 19–25 y age group based on sex. Factors that were significantly associated with increasing PA among male young adults were economic status (*B* = 0.159 and *B* = 0.391), perceived health (*B* = 0.171), happiness (*B* = 0.494), life satisfaction (*B* = 0.188 and *B* = 0.432), and openness (*B* = 0.050). However, having depression (*B* = −0.108) and sleep disturbance (*B* = −0.230) were associated with lower likelihood of having PA. 

Among female young adults, economic status (*B* = 0.217 and *B* = 0.372), perceived health (*B* = 0.156), happiness (*B* = 0.632), and life satisfaction (*B* = 0.174 and *B* = 0.390) were significantly associated with increasing PA. Conversely, living in other islands (*B* = −0.173), living in urban areas (*B* = −0.113), and having depression (*B* = −0.188) were associated with lower likelihood of having PA.

### 3.3. Factors Associated with NA

Table 4 shows the results of the hierarchical multiple linear regression of NA for the 15–18 y group based on sex. Factors that were significantly associated with increasing NA among male adolescents were living in urban areas (*B* = 0.079), acute morbidity symptoms (*B* = 0.177), sleep-related impairment (*B* = 0.203), depression (*B* = 0.273), and openness (*B* = 0.029). However, perceived health (*B* = −0.166) and happiness (*B* = −0.298) were associated with lower likelihood of having NA.

Among female adolescents, acute morbidity symptoms (*B* = 0.156), sleep-related impairment (*B* = 0.304), living with biological mother during childhood (*B* = 0.149), depression (*B* = 0.343), neuroticism (*B* = 0.029), and agreeableness (*B* = 0.029) were significantly associated with increasing NA. Conversely, perceived health (*B* = −0.109), happiness (*B* = −0.514), and living with biological father during childhood (*B* = −0.258) were associated with lower likelihood of having NA.

Table 5 shows the results of the hierarchical multiple linear regression of NA for the 19–25 y group based on sex. Factors that were significantly associated with increasing NA among male young adults were working (*B* = 0.086), acute morbidity symptoms (*B* = 0.187), chronic conditions (*B* = 0.103), sleep-related impairment (*B* = 0.260), depression (*B* = 0.332), neuroticism (*B* = 0.021) and agreeableness (*B* = 0.031). However, economic status (*B* = −0.092 and *B* = −0.107), perceived health (*B* = −0.213) and happiness (*B* = −0.395) were associated with lower likelihood of having NA.

Among female young adults, living in urban areas (*B* = 0.087), smoking cessation (*B* = 0.550), acute morbidity symptoms (*B* = 0.137), sleep-related impairment (*B* = 0.273), depression (*B* = 0.364), conscientiousness (*B* = 0.029), agreeableness (*B* = 0.023), and severity of disasters (*B* = 0.209) were significantly associated with increasing NA. Conversely, perceived health (*B* = −0.178), smoking behavior (*B* = −0.359), happiness (*B* = −0.445), and life satisfaction (*B* = −0.156) were associated with lower likelihood of having NA.

## 4. Discussion

In this study, we aimed to identify sociodemographic characteristics, physical health-related variables, childhood family experiences, and mental health-related variables as determinant factors for PA and NA among adolescents and young adults, and between the two sexes. Several factors were negatively or positively associated with PA and NA. Among adolescents and young adults, PA was positively associated with good economic standing, happiness, life satisfaction, and openness; conversely, it was negatively associated with living in other islands and depression. In addition, among young adults, perceived health was positively associated with PA, and living in urban areas and having sleep disturbance were negatively associated with PA. More disaster experiences were negatively associated with PA in male adolescents. NA was positively associated with living in urban areas, acute morbidity symptoms, sleep-impairment, depression, neuroticism, and agreeableness in adolescents and young adults. Moreover, openness was positively associated with NA in male adolescents. Perceived health and happiness were negatively associated with NA. In female adolescents, living with one’s biological father during childhood was negatively associated with NA, whereas living with one’s biological mother during childhood was positively associated with NA. In male young adults, good economic standing was negatively associated with NA and working status was positively associated with NA. Smoking cessation and conscientiousness were positively associated with NA, whereas smoking behavior was negatively associated with NA among female young adults.

In terms of sociodemographic characteristics, we found that living in an urban area was positively associated with NA among adolescents and young adults. Consistent with this result, Jaya and Wulandari [31] reported that among the general population in Indonesia, participants living in urban areas exhibited higher levels of loneliness, depression, symptoms indicative of psychosis, and negative-self schema. The greater magnitudes of mental health concerns in urban areas compared with those in nonurban areas may be attributable to the differing population densities. Living on other islands was negatively associated with PA among adolescents and young adults. This trend could have been influenced by differences in geographical condition and the distribution of facilities between Java Island and other Indonesian islands. Rejeki, et al. [32] stated that the diverse geographical feature of islands in Indonesia creates challenges for distributing facilities and infrastructure. As the center of the nation’s government, Java possesses some advantages, such as efficient transportation networks, technology, facilities, and infrastructure. Conversely, most other islands, particularly regions in rural Kalimantan, Papua, Sumatra, Sulawesi, and others, are bereft of such advantages. These conditions may also influence individuals’ emotions. Among adolescents and young adults, good economic standing is negatively associated with NA and positively associated with PA. This result accords with previous studies. Abeshi [33] noted that parental socioeconomic status is positively associated with the emotional adjustment of adolescents. Among adults, income is also negatively associated with NA and positively associated with PA [34]. Among male young adults, working status is positively associated with NA. The present study coheres with a previous study from South Korea, which found that in young adults, as the working hours increase, the risk to mental health, such as stress and suicide ideation, tends to increase [35].

For physical health-related variables, we observed that perceived health was associated with higher level of PA in young adults. Moreover, perceived health was negatively associated with NA both in adolescents and young adults. In this study, we discovered that acute morbidity symptoms were positively associated with NA among adolescents and young adults. Few studies have determined acute morbidity symptoms to be predictors of NA among adolescents and young adults. In the general population, somatic symptoms were shown to predict NA [36]. Chronic conditions were identified to be positively associated with NA among male young adults in this study. In this study, adolescents and young adults with sleep-related impairment were more likely to have NA. In line with the present study, Shen, van Schie, Ditchburn, Brook and Bei [21] and Muzni, et al. [37] noted that a lower quality of sleep is associated with higher levels of NA among adolescents and young adults. Sleep disturbance was negatively associated with PA in male adolescents. This agrees with the finding from a previous study that longer sleep duration and good quality of sleep have significant associations with increasing PA in adolescents [21]. The present study found that among female young adults, smoking cessation had a positive association with NA and smoking behavior had a negative association with NA. This is in line with a previous study, wherein difficulty with smoking cessation was higher in female smokers comparing to male smokers. This is because female smokers tended to choose to smoke immediately when they felt a craving and/or stress [38].

In terms of childhood family experiences, we determined that adolescent participants who lived with their biological father during childhood exhibited a greater likelihood of low NA, and those who lived with their biological mother during childhood tended to have higher NA. Few previous studies have identified living with one’s biological father during childhood to be a predictor of NA in adolescents. A longitudinal study discovered that children with more experiences of adverse life events tended to have a higher level of NA when they became adolescents [39].

Furthermore, as regards mental health-related variables, we found that depression was positively associated with NA and negatively associated with PA among adolescents and young adults. Consistent with our result, a previous study indicated that depressive symptoms are associated with decreasing PA in adolescents [40]. However, in this study, we found that greater experience of disasters was negatively associated with increasing PA among adolescents. The significance of this finding is in helping to prevent the negative effects of disasters in adolescents, indicating that coping can be used. Among adolescents, coping can be a factor that protects them from negative outcomes resulting from the traumatic events that they experienced. Coping strategies also can be a link between mass trauma experiences (e.g., natural disasters and manmade disasters) and stress reaction outcomes (positive or negative reaction) [41]. Besides coping, social support is important for adolescents, particularly those who experienced trauma. A study revealed that adolescents with low social support tend to have major depressive disorders [42]. Another study conducted after an earthquake found that social support could reduce PTSD in adolescents [43]. Among female young adults, severity of disasters was positively associated with NA in the present study, in line with previous studies. A study revealed that people who experienced an earthquake were 2.82 time more likely to have depression compared to people who had no experience of an earthquake [44]. Another study reported that the prevalence of depression increased from 8% to 13% after Hurricane Katrina [45].

We also discovered that adolescent and young adult participants with high life satisfaction were more likely to exhibit higher PA and lower NA. This finding is consistent with a previous finding that life satisfaction is significantly and positively associated with PA, and inversely with NA, among adolescents and young adults [19]. This present study also discovered that happiness is positively associated with PA and negatively associated with NA among adolescents and young adults.

We identified that adolescent and young adult participants with higher neuroticism exhibited higher levels of NA, which is consistent with previous studies [46,47]. We also found that agreeableness was positively associated with NA among adolescents and young adults. However, this result does not accord with those of previous studies, which discovered a negative correlation between agreeableness and NA among adolescents and young adults [48,49]. These inconsistencies could be explained by the nature of agreeableness. Agreeableness is the tendency to be cooperative with others, even with someone who might be exploitative [48].

We found that openness was positively associated with PA among adolescents and young adults, a finding that is consistent with previous studies [46,48]. However, in this study’s adolescent participants, openness was also positively associated with NA. Gutiérrez et al. [50] also reported openness to be positively associated with both PA and NA among adults.

This study had several strengths. First, to our best knowledge, this is the first study to investigate the factors associated with PA and NA among adolescents and young adults in Indonesia by surveying a large sample. Second, the characteristics of participants adequately represented those of adolescents and young adults in Indonesia, because the IFLS data were from a national survey, with an 88.95% response rate. However, this study also had some limitations. Because we used self-reported questionnaires, some behaviors may have been underreported or overreported. Furthermore, this study involved secondary data analyses. Thus, we could only assess variables that existed in the selected dataset. Because other variables might also be associated with PA and NA, further pertinent investigations are warranted.

## 5. Conclusions

In this cross-sectional study, we revealed that the distribution of PA and NA between males and females in Indonesia differed among adolescents and young adults. This study provides evidence that determinant factors of PA and NA differed among adolescents and young adults, and between the two sexes. PA is positively associated with better economic standing, perceived health, happiness, life satisfaction, and openness in the two sexes. Furthermore, living in an urban area, working status, acute morbidity symptoms, chronic conditions, depression, sleep-related impairment, openness, neuroticism, agreeableness, smoking cessation, severity of disasters, and living with the biological mother during childhood are significantly associated with increasing NA among adolescents and young adults in Indonesia. The identification of risk factors in this study can be useful in developing future preventive programs.

## Figures and Tables

**Table 1:** Comparison of Personal Characteristics by Age and Gender.

Characteristics	Age Group						*t/X* ^2^
	15–18 y			19–25 y			
	Frequency (%)/Mean (SD)			Frequency (%)/Mean (SD)			
	Male	Female	*t/X* ^2^	Male	Female	*t/X* ^2^	
Marital status							1.700 ***
Unmarried	1385 (98.65)	1346 (89.67)	103.73 ***	1457 (71.14)	996 (37.78)	514.16 ***	
Married	19 (1.35)	155 (10.33)		591 (28.86)	1640 (62.22)		
Attending school							2.608
No	3 (0.21)	1 (0.07)	1.141	7 (0.21)	8 (0.30)	0.053	
Yes	1401 (99.79)	1500 (99.93)		2041 (99.79)	2628 (99.70)		
Economic status							45.992 ***
Poor	223 (15.88)	231 (15.39)	1.032	446 (21.78)	465 (17.64)	21.997 ***	
Middle class	666 (47.44)	692 (46.10)		1041 (50.83)	1304 (49.47)		
Wealthy	515 (36.68)	578 (38.51)		561 (27.39)	867 (32.89)		
Residential area							0.236
Rural	584 (41.60)	566 (37.71)	4.584 *	759 (37.06)	1069 (40.55)	5.911 *	
Urban	820 (58.40)	935 (62.29)		1289 (62.94)	567 (59.45)		
Region							0.883
Java and Bali	801 (57.05)	901 (60.03)	2.647	1186 (57.91)	1507 (57.17)	0.258	
Other Islands	603 (42.95)	600 (39.97)		862 (42.09)	1129 (42.83)		
Work status							1.300 ***
No	1171 (83.40)	1345 (89.61)	24.063 ***	571 (27.88)	1647 (62.48)	553.47 ***	
Yes	233 (16.60)	156 (10.39)		1477 (72.12)	989 (37.52)		
Perceived health							12.585 ***
Unhealthy	175 (12.46)	219 (14.59)	2.797	286 (13.96)	490 (18.59)	17.828 ***	
Healthy	1229 (87.54)	1282 (85.41)		1762 (86.04)	2146 (81.41)		
Smoking habit							217.74 ***
Nonsmoking	934 (66.52)	1497 (99.73)	585.93 ***	624 (30.47)	2600 (98.63)	2.500 ***	
Smoking	470 (33.48)	4 (0.27)		1424 (69.53)	36 (1.37)		
Smoking cessation							212.03 ***
Nonsmoker	974 (69.37)	1500 (99.93)	536.20 ***	705 (34.42)	2616 (99.24)	2.300 ***	
Current smoker	430 (30.63)	1 (0.07)		1343 (65.58)	20 (0.76)		
Acute morbidities							3.725
0 symptoms	349 (24.86)	232 (15.46)	40.073 ***	446 (21.78)	407 (15.44)	31.077 ***	
≥ 1 symptom(s)	1055 (75.14)	1269 (84.54)		1602 (78.22)	2229 (84.56)		
Chronic conditions							22.494 ***
No	1173 (83.55)	1148 (76.48)	22.542 ***	1635 (79.83)	1888 (71.62)	41.674 ***	
Yes	231 (16.45)	353 (23.52)		413 (20.17)	748 (28.38)		
Sleep disturbance							18.758 ***
No	846 (97.02)	958 (96.28)	0.774	1334 (93.16)	1604 (94.58)	2.740	
Yes	26 (2.98)	37 (3.72)		98 (6.84)	92 (5.42)		
Sleep-related impairment							0.887
No	663 (76.03)	784 (78.79)	2.033	1073 (74.93)	1315 (77.54)	2.918	
Yes	209 (23.97)	211 (21.21)		359 (25.07)	381 (22.46)		
Depression							3.118
No	1053 (75.00)	1004 (66.89)	23.091 ***	1369 (66.85)	1858 (70.49)	7.125 **	
Yes	351 (25.00)	497 (33.11)		679 (33.15)	778 (29.51)		
Personality							
Extraversion	10.42 (±1.62)	10.56 (±1.54)	−2.476 *^a^	10.52 (±1.57)	10.32 (±1.54)	4.272 ^a^	2.366 *^a^
Conscientiousness	10.22 (±1.63)	10.57 (±1.63)	−5.915 ***^a^	10.43 (±1.57)	10.45 (±1.53)	−0.445 ^a^	−1.092 ^a^
Openness	11.41 (±1.91)	11.47 (±1.86)	−0.874 ^a^	11.53 (±1.86)	11.08 (±1.87)	8.062 ***^a^	3.733 ***^a^
Neuroticism	9.97 (±1.98)	10.47 (±2.01)	−6.779 ***^a^	10.19 (±2.00)	10.35 (±1.96)	−2.725 **^a^	−1.226 ^a^
Agreeableness	11.23 (±1.54)	11.36 (±1.57)	−2.360 *^a^	11.14 (±1.53)	10.93 (±1.50)	4.674 ***^a^	7.624 ***^a^
Life satisfaction							27.729 ***
Unsatisfied	109 (7.76)	141 (9.39)	3.647	295 (14.40)	262 (9.94)	25.101 ***	
Somewhat satisfied	546 (38.89)	602 (40.11)		843 (41.16)	1075 (40.78)		
Satisfied	749 (53.35)	758 (50.50)		910 (44.43)	1299 (49.28)		
Happiness							0.020
Unhappy	78 (5.56)	83 (5.53)	0.001	146 (7.13)	110 (4.17)	19.491 ***	
Happy	1326 (94.44)	1418 (94.47)		1902 (92.87)	2526 (95.83)		
Disaster experiences							0.047
No	1126 (80.20)	1155 (76.95)	4.545 *	1599 (78.08)	2069 (78.49)	0.116	
Yes	278 (19.80)	346 (23.05)		449 (21.92)	567 (21.51)		
Severe disaster experiences							0.438
No	1368 (97.44)	1452 (96.74)	1.253	1993 (97.31)	2566 (97.34)	0.004	
Yes	36 (2.56)	49 (3.26)		55 (2.69)	70 (2.66)		
Self-rated childhood health							18.802 ***
Unhealthy	445 (31.70)	469 (31.25)	0.068	726 (35.45)	975 (36.99)	1.179	
Healthy	959 (68.30)	1032 (68.75)		1322 (64.55)	1661 (63.01)		
Biological parents still married during childhood							4.362 *
No	194 (13.82)	237 (15.79)	2.232	254 (12.40)	361 (13.69)	1.688	
Yes	1210 (86.18)	1264 (84.21)		1794 (87.60)	2275 (86.31)		
Living with biological mother during childhood							13.155 ***
No	165 (11.75)	223 (14.86)	6.042 *	203 (9.91)	293 (11.12)	0.184	
Yes	1239 (88.25)	1278 (85.14)		1845 (90.09)	2343 (88.88)		
Living with biological father during childhood							12.364 ***
No	246 (17.52)	320 (21.32)	6.669 *	316 (15.43)	448 (17.00)	2.070	
Yes	1158 (37.37)	1181 (78.68)		1732 (37.37)	2188 (83.00)		
Living with grandparents during childhood							7.125 **
No	1038 (73.93)	1073 (71.49)	2.185	1531 (74.76)	2002 (75.95)	0.884	
Yes	366 (26.07)	428 (28.51)		517 (25.24)	634 (24.05)		
Positive affects	3.27 (±1.00)	3.13 (±0.98)	3.642 ***^a^	3.15 (±1.02)	3.09 (±1.00)	1.974 *^a^	3.340 ***^a^
Negative affects	1.66 (±0.59)	1.79 (±0.67)	−5.571 ***^a^	1.78 (±0.70)	1.75 (±0.67)	1.459 ^a^	−2.450 *^a^

Note: * *p* < 0.05; ** *p* < 0.01; *** *p* < 0.001; ^a^ Independent T-test.

**Table 2:** Hierarchical multiple linear regression on positive affect by associated psychosocial and health-related factors in the 15–18 y age group based on sex.

Variables	Male				Female			
	Step 1	Step 2	Step 3	Step 4	Step 1	Step 2	Step 3	Step 4
	*B*	*B*	*B*	*B*	*B*	*B*	*B*	*B*
Sociodemographic Characteristics								
Marital Status								
Unmarried	As reference				As reference			
Married	−0.456	−0.442	−0.473	−0.378	0.011	0.020	0.023	0.058
Attending school								
No	As reference				As reference			
Yes	0.487	0.498	0.526	0.702	0.454	0.498	0.503	0.419
Economic status								
Poor	As reference				As reference			
Middle class	0.192 *	0.172	0.199 *	0.125	0.143	0.126	0.123	0.064
Wealthy	0.415 ***	0.376 ***	0.380 ***	0.227 *	0.254 **	0.222 *	0.218 *	0.085
Residential area								
Rural	As reference				As reference			
Urban	0.117	0.119	0.101	0.098	0.053	0.041	0.038	0.028
Region								
Java and Bali	As reference				As reference			
Other islands	−0.138 *	−0.129	−0.147 *	−0.166 *	−0.156 *	−0.148 *	−0.148 *	−0.188 **
Work status								
No	As reference				As reference			
Yes	−0.148	−0.116	−0.094	−0.076	−0.051	−0.034	−0.024	0.019
Health-related variables								
Perceived health								
Unhealthy	As reference				As reference			
Healthy		0.174	0.172	0.052		0.276 **	0.269 **	0.139
Smoking habit								
Non-smoking	As reference				As reference			
Smoking		−0.110	−0.097	0.002		−0.515	−0.470	−0.384
Smoking cessation								
Non-smoker	As reference				As reference			
Current smoker		0.043	0.029	0.005		2.176 *	2.091	1.936
Acute morbidities								
0 symptom	As reference				As reference			
≥1 symptom(s)		−0.034	−0.030	0.005		0.065	0.067	0.102
Chronic conditions								
No	As reference				As reference			
Yes		0.104	0.121	0.084		0.094	0.096	0.128
Sleep disturbance								
No	As reference				As reference			
Yes		−0.043	−0.060	−0.009		−0.275	−0.279	−0.299
Sleep-related impairment								
No	As reference				As reference			
Yes		0.021	0.020	0.065		−0.137	−0.140	−0.128
Childhood family experiences								
Self-rated childhood health								
Unhealthy	As reference				As reference			
Healthy			0.172 *	0.103			0.055	0.007
Biological parents still married during childhood								
No	As reference				As reference			
Yes			−0.239	−0.243			−0.001	0.024
Living with biological mother during childhood								
No	As reference				As reference			
Yes			−0.120	−0.116			−0.013	0.015
Living with biological father during childhood								
No	As reference				As reference			
Yes			0.141	0.166			0.097	0.003
Living with grandparents during childhood								
No	As reference				As reference			
Yes			−0.077	−0.117			−0.008	−0.018
Mental health-related variables								
Depression								
No	As reference				As reference			
Yes				−0.160 *				−0.120
Personality								
Extraversion				0.031				−0.012
Conscientiousness				0.037				−0.012
Openness				0.062 **				0.065 ***
Neuroticism				−0.045 *				0.003
Agreeableness				−0.026				0.035
Life satisfaction								
Unsatisfied	As reference				As reference			
Somewhat satisfied				0.155				0.184
Satisfied				0.393 **				0.509 ***
Happiness								
Unhappy	As reference				As reference			
Happy				0.560 ***				0.457 ***
Disaster experiences								
No	As reference				As reference			
Yes				−0.189 *				0.037
Severe disaster experiences								
No	As reference				As reference			
Yes				0.354				0.058
R2	0.049	0.055	0.070	0.160	0.017	0.041	0.044	0.117
R2 Change	0.049	0.007	0.015	0.090	0.017	0.024	0.002	0.073
*p*	0.000	0.525	0.019	0.000	0.016	0.001	0.816	0.000

Note: * *p* < 0.05; ** *p* < 0.01; *** *p* < 0.001.

**Table 3:** Hierarchical multiple linear regression on positive affect by associated psychosocial and health related-factors in the 19–25 y age group based on sex.

Variables	Male				Female			
	Step 1	Step 2	Step 3	Step 4	Step 1	Step 2	Step 3	Step 4
	*B*	*B*	*B*	*B*	*B*	*B*	*B*	*B*
Sociodemographic characteristics								
Marital status								
Unmarried	As reference				As reference			
Married	0.081	0.075	0.078	0.054	−0.039	−0.036	−0.038	−0.018
Attending school								
No	As reference				As reference			
Yes	0.447	0.395	0.336	0.382	0.685	0.613	0.628	0.593
Economic status								
Poor	As reference				As reference			
Middle class	0.308 ***	0.300 ***	0.292 ***	0.195 **	0.330 ***	0.316 ***	0.314 ***	0.217 **
Wealthy	0.639 ***	0.609 ***	0.588 ***	0.391 ***	0.566 ***	0.544 ***	0.537 ***	0.372 ***
Residential area								
Rural	As reference				As reference			
Urban	0.024	0.021	0.028	0.019	−0.115	−0.107 *	−0.111 *	−0.113 *
Region								
Java and Bali	As reference				As reference			
Other islands	−0.084	−0.077	−0.085	−0.083	−0.175 ***	−0.161 **	−0.162 **	−0.173 **
Work status								
No	As reference				As reference			
Yes	0.120 *	0.098	0.103	0.070	0.039	0.048	0.047	0.056
Health-related variables								
Perceived health								
Unhealthy	As reference				As reference			
Healthy		0.240 **	0.242 **	0.171 *		0.235 ***	0.232 ***	0.156 **
Smoking habit								
Non-smoking	As reference				As reference			
Smoking		0.060	0.067	0.081		−0.038	−0.025	0.149
Smoking cessation								
Non-smoker	As reference				As reference			
Current smoker		−0.070	−0.074	−0.081		−0.209	−0.220	−0.356
Acute morbidities								
0 symptom	As reference				As reference			
≥1 symptom(s)		0.131	0.138 *	0.130		0.071	0.075	0.087
Chronic conditions								
No	As reference				As reference			
Yes		−0.122	−0.104	−0.103		−0.040	−0.037	−0.006
Sleep disturbance								
No	As reference				As reference			
Yes		−0.228 *	−0.239 *	−0.230 *		−0.082	−0.077	−0.017
Sleep-related impairment								
No	As reference				As reference			
Yes		0.036	0.055	0.100		−0.008	−0.010	0.039
Childhood family experiences								
Self-rated childhood health								
Unhealthy	As reference				As reference			
Healthy			0.082	0.048			0.057	0.018
Biological parents still married during childhood								
No	As reference				As reference			
Yes			0.148	0.149			−0.074	−0.069
Living with biological mother during childhood								
No	As reference				As reference			
Yes			−0.128	−0.123			0.039	0.012
Living with biological father during childhood								
No	As reference				As reference			
Yes			0.107	0.099			0.056	0.049
Living with grandparents during childhood								
No	As reference				As reference			
Yes			−0.089	−0.074			−0.046	−0.043
Mental health-related variables								
Depression								
No	As reference				As reference			
Yes				−0.108 *				−0.188 ***
Personality								
Extraversion				0.010				0.029
Conscientiousness				0.013				0.008
Openness				0.050 **				0.023
Neuroticism				−0.025				−0.002
Agreeableness				0.032				0.013
Life satisfaction								
Unsatisfied	As reference				As reference			
Somewhat satisfied				0.188 *				0.174 *
Satisfied				0.432 ***				0.390 ***
Happiness								
Unhappy	As reference				As reference			
Happy				0.494 ***				0.632 ***
Disaster experiences								
No	As reference				As reference			
Yes				0.105				0.047
Severe disaster experiences								
No	As reference				As reference			
Yes				0.027				−0.247
R2	0.053	0.070	0.078	0.151	0.051	0.063	0.065	0.131
R2 Change	0.053	0.017	0.008	0.073	0.051	0.012	0.002	0.066
*p*	0.000	0.001	0.037	0.000	0.000	0.003	0.677	0.000

Note: * *p* < 0.05; ** *p* < 0.01; *** *p* < 0.001.

**Table 4:** Hierarchical multiple linear regression on negative affect by psychosocial and health-related factors in the 15–18 y age group based on sex.

Variables	Male				Female			
	Step 1	Step 2	Step 3	Step 4	Step 1	Step 2	Step 3	Step 4
	*B*	*B*	*B*	*B*	*B*	*B*	*B*	*B*
Sociodemographic characteristics								
Marital status								
Unmarried	As reference				As reference			
Married	0.038	−0.026	−0.016	−0.083	−0.078	−0.084	−0.085	−0.073
Attending school								
No	As reference				As reference			
Yes	−0.141	−0.240	−0.223	−0.326	−0.261	−0.700	−0.919	−0.775
Economic status								
Poor	As reference				As reference			
Middle class	−0.095	−0.074	−0.079	−0.076	−0.243 ***	−0.209 **	−0.203 **	−0.112
Wealthy	−0.204 **	−0.153 *	−0.150 *	−0.112	−0.161 *	−0.115	−0.112	−0.278
Residential area								
Rural	As reference				As reference			
Urban	0.082	0.062	0.063	0.079 *	0.069	0.024	0.025	0.035
Region								
Java and Bali	As reference				As reference			
Other islands	−0.041	−0.047	−0.044	−0.025	−0.016	−0.007	−0.014	−0.004
Work status								
No	As reference				As reference			
Yes	0.066	0.044	0.035	0.058	0.109	0.063	0.060	0.049
Health-related variables								
Perceived health								
Unhealthy	As reference				As reference			
Healthy		−0.238 ***	−0.233 ***	−0.166 **		−0.277 ***	−0.267 ***	−0.109 *
Smoking habit								
Non-smoking	As reference				As reference			
Smoking		0.008	−0.019	−0.055		−0.448	−0.488	−0.488
Smoking cessation								
Non-smoker	As reference				As reference			
Current smoker		−0.048	−0.028	−0.005		−0.076	0.298	0.543
Acute morbidities								
0 symptom	As reference				As reference			
≥ 1 symptom(s)		0.202 ***	0.199 ***	0.177 ***		0.260 ***	0.258 ***	0.156 *
Chronic conditions								
No	As reference				As reference			
Yes		0.076	0.072	0.061		0.085	0.089	0.089
Sleep disturbance								
No	As reference				As reference			
Yes		0.299 *	0.293 *	0.195		0.096	0.134	0.078
Sleep-related impairment								
No	As reference				As reference			
Yes		0.303 ***	0.299 ***	0.203 ***		0.430 ***	0.438 ***	0.304 ***
Childhood family experiences								
Self-rated childhood health								
Unhealthy	As reference				As reference			
Healthy			−0.064	−0.026			−0.046	−0.011
Biological parents still married during childhood								
No	As reference				As reference			
Yes			−0.004	−0.002			0.092	0.123
Living with biological mother during childhood								
No	As reference				As reference			
Yes			−0.008	−0.015			0.150	0.149 *
Living with biological father during childhood								
No	As reference				As reference			
Yes			−0.093	−0.106			−0.259 **	−0.258 **
Living with grandparents during childhood								
No	As reference				As reference			
Yes			−0.028	−0.037			−0.082	−0.073
Mental health-related variables								
Depression								
No	As reference				As reference			
Yes				0.273 ***				0.343 ***
Personality								
Extraversion				−0.005				0.010
Conscientiousness				−0.012				0.005
Openness				0.029 *				0.013
Neuroticism				0.020				0.029 **
Agreeableness				0.018				0.029 *
Life satisfaction								
Unsatisfied	As reference				As reference			
Somewhat satisfied				−0.056				−0.014
Satisfied				−0.102				−0.100
Happiness								
Unhappy	As reference				As reference			
Happy				−0.298 ***				−0.514 ***
Disaster experiences								
No	As reference				As reference			
Yes				−0.006				0.058
Severe disaster experiences								
No	As reference				As reference			
Yes				−0.088				0.012
R2	0.023	0.135	0.141	0.227	0.021	0.144	0.157	0.284
R2 Change	0.023	0.112	0.006	0.086	0.021	0.124	0.013	0.127
*p*	0.005	0.000	0.306	0.000	0.004	0.000	0.012	0.000

Note: * *p* < 0.05; ** *p* < 0.01; *** *p* < 0.001.

**Table 5:** Hierarchical multiple linear regression on negative affect by psychosocial and health-related factors in the 19–25 y age group based on sex.

Variables	Male				Female			
	Step 1	Step 2	Step 3	Step 4	Step 1	Step 2	Step 3	Step 4
	*B*	*B*	*B*	*B*	*B*	*B*	*B*	*B*
Sociodemographic characteristics								
Marital status								
Unmarried	As reference				As reference			
Married	−0.096 *	−0.094 *	−0.092 *	−0.051	−0.082 **	−0.072 *	−0.067 *	−0.049
Attending school								
No	As reference				As reference			
Yes	0.578	0.456	0.465	0.325	0.502	0.449	0.437	0.399
Economic status								
Poor	As reference				As reference			
Middle class	−0.150 **	−0.131 **	−0.131 **	−0.092 *	−0.075	−0.046	−0.046	0.033
Wealthy	−0.244 ***	−0.194 ***	−0.193 ***	−0.107 *	−0.211 ***	−0.172 ***	−0.171 ***	−0.043
Residential area								
Rural	As reference				As reference			
Urban	0.047	0.026	0.024	0.039	0.110 **	0.073 *	0.072 *	0.087 **
Region								
Java and Bali	As reference				As reference			
Other islands	−0.075	−0.078 *	−0.077 *	−0.069	0.015	0.014	0.016	0.004
Work status								
No	As reference				As reference			
Yes	0.063	0.071	0.071	0.086 *	−0.002	−0.002	−0.003	−0.026
Health-related variables								
Perceived health								
Unhealthy	As reference				As reference			
Healthy		−0.301 ***	−0.304 ***	−0.213 ***		−0.257 ***	−0.258 ***	−0.178 ***
Smoking habit								
Non-smoking	As reference				As reference			
Smoking		0.000	0.002	−0.077		−0.143	−0.146	−0.359*
Smoking cessation								
Non-smoker	As reference				As reference			
Current smoker		−0.068	−0.071	−0.028		0.316	0.324	0.550 *
Acute morbidities								
0 symptom	As reference				As reference			
≥ 1 symptom(s)		0.224 ***	0.223 ***	0.187 ***		0.191 ***	0.194 ***	0.137 **
Chronic conditions								
No	As reference				As reference			
Yes		0.142 **	0.137 **	0.103 *		0.093 **	0.094 **	0.058
Sleep disturbance								
No	As reference				As reference			
Yes		0.112	0.116	0.020		0.167 *	0.158 *	0.083
Sleep-related impairment								
No	As reference				As reference			
Yes		0.386 ***	0.382 ***	0.260 ***		0.377 ***	0.379 ***	0.273 ***
Childhood family experiences								
Self-rated childhood health								
Unhealthy	As reference				As reference			
Healthy			0.002	0.020			0.001	0.011
Biological parents still married during childhood								
No	As reference				As reference			
Yes			0.054	0.065			0.095	0.093
Living with biological mother during childhood								
No	As reference				As reference			
Yes			0.008	−0.046			−0.054	−0.026
Living with biological father during childhood								
No	As reference				As reference			
Yes			−0.105	−0.091			−0.043	−0.046
Living with grandparents during childhood								
No	As reference				As reference			
Yes			0.000	−0.032			0.057	0.051
Mental health-related variables								
Depression								
No	As reference				As reference			
Yes				0.332 ***				0.364 ***
Personality								
Extraversion				0.001				0.013
Conscientiousness				−0.008				0.029 **
Openness				0.016				−0.009
Neuroticism				0.021 *				0.008
Agreeableness				0.031 *				0.023 *
Life satisfaction								
Unsatisfied	As reference				As reference			
Somewhat satisfied				−0.001				−0.061
Satisfied				−0.090				−0.156 **
Happiness								
Unhappy	As reference				As reference			
Happy				−0.395 ***				−0.445 ***
Disaster experiences								
No	As reference				As reference			
Yes				0.038				−0.018
Severe disaster experiences								
No	As reference				As reference			
Yes				−0.030				0.209 *
R2	0.021	0.151	0.152	0.253	0.023	0.133	0.136	0.248
R2 Change	0.021	0.130	0.001	0.101	0.023	0.110	0.003	0.113
*p*	0.000	0.000	0.824	0.000	0.000	0.000	0.358	0.000

Note: * *p* < 0.05; ** *p* < 0.01; *** *p* < 0.001.

## Data Availability

Not applicable.
